# Enhanced Responses to Tumor Immunization Following Total Body Irradiation Are Time-Dependent

**DOI:** 10.1371/journal.pone.0082496

**Published:** 2013-12-12

**Authors:** Adi Diab, Robert R. Jenq, Gabrielle A. Rizzuto, Adam D. Cohen, Deonka W. Huggins, Taha Merghoub, Manuel E. Engelhorn, José A. Guevara-Patiño, David Suh, Vanessa M. Hubbard-Lucey, Adam A. Kochman, Suzie Chen, Hong Zhong, Jedd D. Wolchok, Marcel R. M. van den Brink, Alan N. Houghton, Miguel-Angel Perales

**Affiliations:** 1 Swim Across America Laboratory of Tumor Immunology, Department of Medicine, Memorial Sloan-Kettering Cancer Center, New York, New York, United States of America; 2 Adult Bone Marrow Transplantation Service, Department of Medicine, Memorial Sloan-Kettering Cancer Center, New York, New York, United States of America; 3 Immunology Program, Sloan-Kettering Institute, New York, New York, United States of America; 4 Weill Cornell Medical College, New York, New York, United States of America; 5 Susan Lehman Cullman Laboratory for Cancer Research, Ernest Mario School of Pharmacy, Rutgers, The State University of New Jersey Piscataway, New Brunswick, New Jersey, United States of America; Mie University Graduate School of Medicine, Japan

## Abstract

The development of successful cancer vaccines is contingent on the ability to induce effective and persistent anti-tumor immunity against self-antigens that do not typically elicit immune responses. In this study, we examine the effects of a non-myeloablative dose of total body irradiation on the ability of tumor-naïve mice to respond to DNA vaccines against melanoma. We demonstrate that irradiation followed by lymphocyte infusion results in a dramatic increase in responsiveness to tumor vaccination, with augmentation of T cell responses to tumor antigens and tumor eradication. In irradiated mice, infused CD8^+^ T cells expand in an environment that is relatively depleted in regulatory T cells, and this correlates with improved CD8^+^ T cell functionality. We also observe an increase in the frequency of dendritic cells displaying an activated phenotype within lymphoid organs in the first 24 hours after irradiation. Intriguingly, both the relative decrease in regulatory T cells and increase in activated dendritic cells correspond with a brief window of augmented responsiveness to immunization. After this 24 hour window, the numbers of dendritic cells decline, as does the ability of mice to respond to immunizations. When immunizations are initiated within the period of augmented dendritic cell activation, mice develop anti-tumor responses that show increased durability as well as magnitude, and this approach leads to improved survival in experiments with mice bearing established tumors as well as in a spontaneous melanoma model. We conclude that irradiation can produce potent immune adjuvant effects independent of its ability to induce tumor ablation, and that the timing of immunization and lymphocyte infusion in the irradiated host are crucial for generating optimal anti-tumor immunity. Clinical strategies using these approaches must therefore optimize such parameters, as the correct timing of infusion and vaccination may mean the difference between an ineffective treatment and successful tumor eradication.

## Introduction

Developing vaccines to prevent or treat malignancy represents an appealing strategy that could potentially be combined with conventional treatments. The major challenges in developing effective vaccine therapy against cancer have been surmounting the barriers which prevent development of immune responses against self-antigens as well as mechanisms by which tumors can induce immune ignorance or tolerance [1]. As summarized by Klebanoff et al, results of most clinical trials of cancer vaccines have not shown a clinical benefit, despite the ability of many vaccines to produce measurable immune responses [2]. However, clinical progress has recently been accelerating, with three Phase 3 clinical trials demonstrating a survival benefit with vaccine therapies directed against lymphoma, melanoma, and prostate cancer [3-5]. These results affirm that cancer vaccines have an emerging role to play in the management of malignancy.

One potential strategy to enhance cancer vaccines is not to build a better vaccine, but to instead utilize established vaccine approaches and combine them with strategies to improve the ability of individuals to respond to tumor immunization. Inducing lymphopenia with irradiation may be such an approach, having already been demonstrated to augment adoptive T cell therapy of cancer [6-11]. Dummer et al. [7] showed that the transfer of naïve T cells into sublethally irradiated mice could slow tumor growth, through the expansion of polyclonal tumor-specific CD8^+^ T cells. A second group confirmed these results and also demonstrated an increase in the percentage of T cells expressing an activated CD44^hi^CD62L^lo^ phenotype in irradiated mice [8]. Subsequent studies demonstrated increased availability of pro-survival and activating cytokines including IL-7 and IL-15 in the lymphopenic environment [10], while others have shown reduced numbers of regulatory T cells [12], and a reduced threshold of activation and expansion of self-reactive T cell clones, which results in a beneficial anti-tumor response [13]. Given the well-established ability of irradiation to augment adoptive T cell therapies, in this study we have hypothesized that irradiation would similarly augment immune responses to a T cell cancer vaccine. Our results support this hypothesis. We observe increased frequency of tumor specific CD8+ T cells, augmented tumor protection and eradication in mice treated with combination therapy of irradiation, lymphocyte infusion, and vaccination when compared to single or dual-therapy. The enhancement is exquisitely sensitive to the timing of irradiation and vaccination. Efficacy correlates with the presence of activated dendritic cells that presumably prime the observed larger population of vaccine-generated tumor antigen specific CD8+ T cells.

## Results

### Irradiation followed by naïve lymphocyte infusion enhances T cell responses to tumor immunization

**Figure 1:**
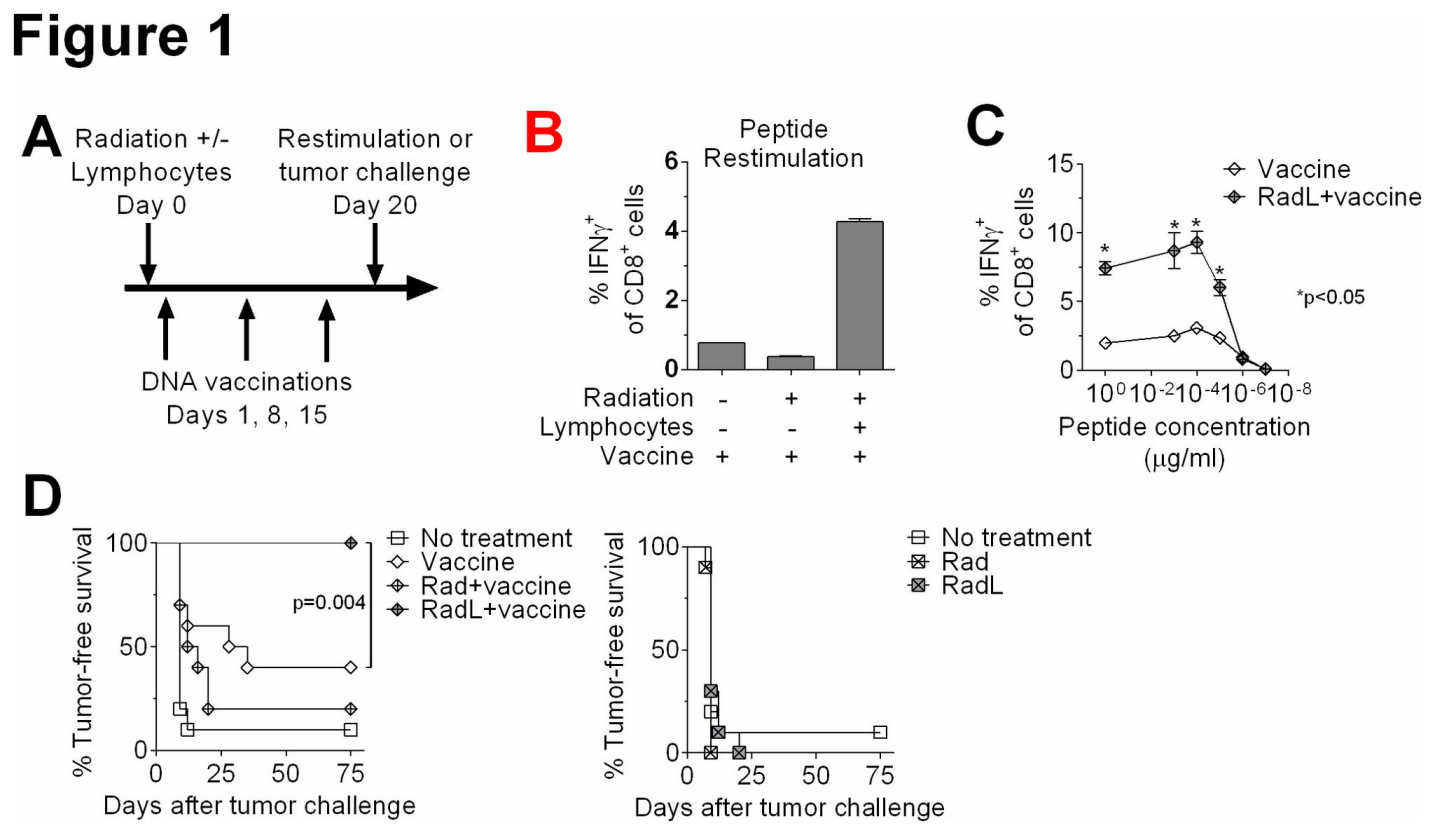
Irradiation followed by lymphocyte infusion leads to augmented responses to tumor vaccination. A) Schematic of experimental protocol. Mice were immunized with plasmid DNA against melanoma antigens for 3 weekly doses. One day prior to the first immunization some mice received 6 Gy total body irradiation (Rad) or irradiation followed by lymphocyte infusion (30×10^6^ splenocytes from naïve mice, RadL). B) Mice were treated as in A. After hTRP2 DNA immunizations, splenocytes were restimulated with TRP2_181-188_ peptide (1μg/ml) and IFNγ production from CD8^+^ T cells was quantified by flow cytometry. n=3/group, results shown from one of two experiments with similar results. C) Mice were treated as in A. After VP22-Opt-TRP1 DNA immunizations, splenocytes were restimulated with the indicated concentrations of TRP1_455-463_ peptide [17] and IFNγ production from CD8^+^ T cells was quantified by flow cytometry. n=3/group. D) Mice were treated as in B, and then after hTRP2 DNA immunizations were challenged intradermally with B16 melanoma. Mice were monitored for development of palpable tumors. n=10/group, results shown from one of three experiments with similar results.

While lymphopenia has been demonstrated to enhance anti-tumor immune responses in a variety of settings, many of these prior studies utilized mice with genetic enhancement of T cell tumor specificity [11], or mice with genetically-induced absence of lymphocytes [14]. In this study, we have focused on clinically relevant mouse models, making use of mice with normal T cell repertoires in all tumor experiments. We also chose to examine the effects of sublethal total body irradiation (6 Gy), a clinically translatable inducer of lymphopenia. Finally, we treated mice with DNA vaccines against melanoma that are murine analogues of those in clinical trials [15]. We began by studying the effects of irradiation on the efficacy of two different vaccines that immunize against the melanocyte differentiation antigens TRP2 and TRP1, respectively. We administered plasmid DNA encoding human TRP2 (hTRP2) [16] or murine TRP1 mutated for optimized MHC class I binding and fused to the herpes simples virus type 2 VP22 protein (VP22-Opt-TRP1) [17]. Some mice received irradiation without or with lymphocyte infusion prior to immunizations (Figure 1A).

We found that irradiation prior to vaccination resulted in loss of responsiveness to immunizations, demonstrated by a reduction in T cell responses to peptide restimulation (Figure 1B) as well as an inferior ability to reject an intradermal challenge with murine B16 melanoma (Figure 1D). However, rescue of irradiated mice with an infusion of unmanipulated splenocytes prior to vaccination resulted in enhanced T cell responses to immunization with both TRP2 (Figure 1B) and VP22-Opt-TRP1 (Figure 1C). Radiation and lymphocyte infusion also improved the ability of vaccinated mice to reject B16 melanoma with 100% tumor rejection, compared to 40% in unirradiated, vaccinated mice (Figure 1D). Using congenic markers to distinguish residual lymphocytes that had survived irradiation from infusion-derived T cells, we found that vaccine-specific CD8^+^ T cells after irradiation were all of infusion origin (data not shown). Stimulating T cells with varying peptide concentrations demonstrated that radiation resulted in enhanced numbers of both lower and higher affinity T cells (Figure 1C). 

### Irradiation followed by naïve lymphocyte infusion results in lymphopenia with an increased CD8^+^ to regulatory T cell ratio and improved CD8^+^ T cell function

**Figure 2:**
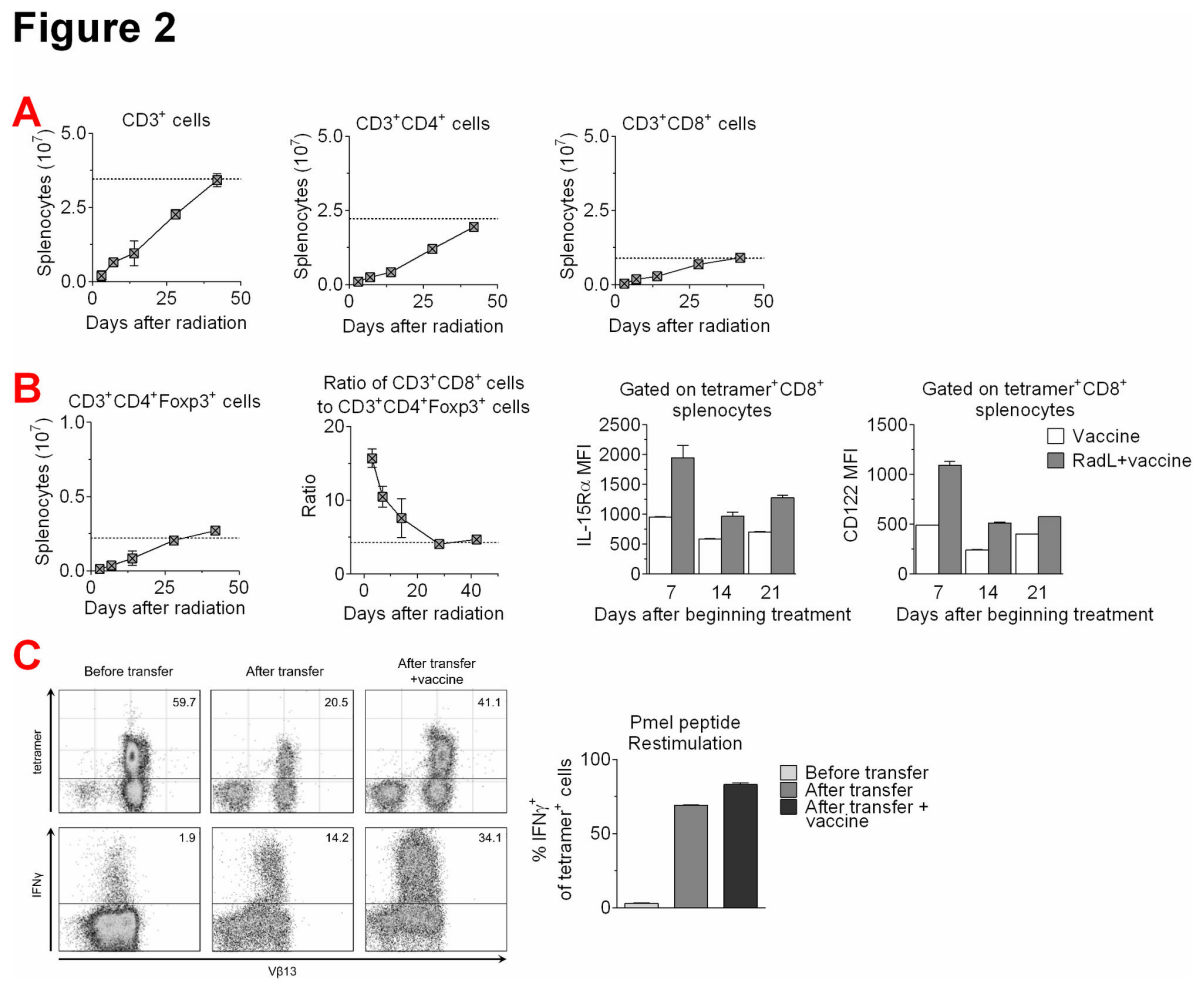
Irradiation followed by lymphocyte infusion leads to marked T cell populations and increased frequency of IFNy+ tumor-antigen specific CD8+ T cells. A) CD3, CD4 and CD8-expressing splenocytes were quantified by flow cytometry at the indicated time points following irradiation and lymphocyte infusion. Typical numbers from normal mice are indicated by the dotted line. Results shown from one of two experiments with similar results. B) Regulatory T cell splenocytes (CD3^+^CD4^+^Foxp3^+^) and the CD8^+^ T cell to regulatory T cell ratio were quantified by flow cytometry. Expression of IL-15Rα and CD122 on tetramer^+^ CD8^+^ T cells was also quantified by flow cytometry. Results shown from one of two experiments with similar results. C) Mice were irradiated, received an infusion of 30×10^6^ splenocytes from naïve Pmel mice, which express a CD8^+^ TCR transgene recognizing the melanoma antigen gp100, and immunized weekly with hgp100 DNA plasmid vaccine for 3 doses. Naive Pmel splenocytes, and splenocytes isolated 20 days after infusion into irradiated animals +/- vaccination were restimulated with gp100_25-33_ peptide (1μg/ml) and evaluated for staining with Pmel-specific tetramer and expression of IFNγ by flow cytometry.

To explore why radiation and lymphocyte infusion enhances responses to immunization, we characterized T cell expansion following infusion. It takes up to 6 weeks for T cell numbers to return to normal levels, with similar recovery rates for total T cells, CD4^+^ T cells, and CD8^+^ T cells (Figure 2A). At early time points, CD8^+^ T cells were entirely of infusion origin, whereas beyond 3 weeks some CD8^+^ T cells were endogenously derived from the irradiated mouse (data not shown). In contrast, CD4^+^ T cells were significantly more radio-resistant, with only ~30% of CD4^+^ T cells of infusional origin even at early time points (data not shown). We also evaluated numbers of regulatory T cells characterized by expression of CD4 and the transcription factor Foxp3. We found that all T cell subsets, including regulatory T cells, gradually recovered their numbers over 6 weeks (Figure 2B). At early time points, however, CD8^+^ T cell numbers displayed an accelerated pace of recovery compared to regulatory T cells, leading to a 3-fold increase in the ratio of CD8^+^ T cells to regulatory T cells on the first day after infusion (Figure 2B). As noted above, the antigen-specific CD8^+^ T cells were derived from the infusion, indicating this ratio may be critical to the early enhanced T cell responses we observed. Supporting a proliferative advantage of CD8^+^ T cells, we found that CD8^+^ T cells infused after irradiation upregulated components of the IL-15 receptor, including IL-15Rα and CD122 (Figure 2B).

Given this evidence suggesting that effector CD8^+^ T cells may be receiving reduced regulatory signals following radiation, we asked if they would increase their acquisition of effector functions in the post-radiation environment. We utilized the Pmel-1 mouse, which overexpresses a CD8^+^ T cell receptor (TCR) specific for the melanoma antigen gp100 [18]. Upon stimulation with gp100 peptide, IFNγ is produced by only a very low frequency of naïve Pmel T cells (Figure 2C). Twenty days after transfer to an irradiated mouse, however, the majority of TCR transgenic cells produce IFNγ upon ex vivo restimulation. We found that immunization with human gp100 plasmid DNA [19] is not necessary for this improved functionality, though the addition of immunization does lead to a further increase in the frequency of cytokine-producing CD8^+^ T cells (Figure 2C). Together, these results demonstrate that irradiation followed by lymphocyte infusion augments the frequency of vaccine-generated tumor antigen specific, IFNγ-producing CD8+ T cells. This enhanced effect correlates with a relative depletion of CD4+Foxp3+ T cells at the time of vaccination.

### Radiation results in a short-lived enhancement of dendritic cell numbers and phenotype, corresponding to a short window of enhanced responsiveness to tumor vaccination

**Figure 3:**
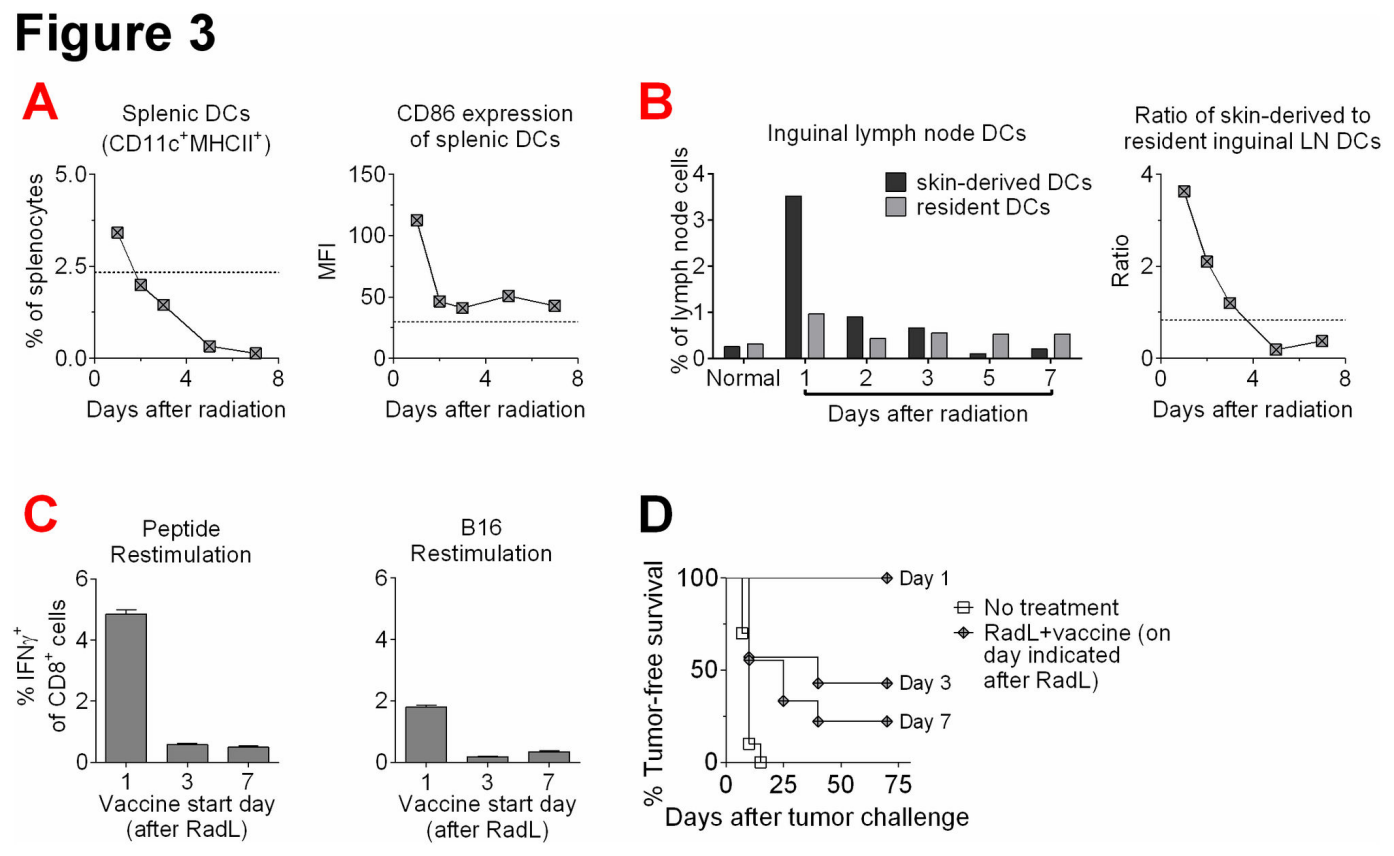
Irradiation results in a short-lived enhancement of dendritic cell numbers and phenotype, corresponding to a similarly short-lived enhanced response to tumor vaccination. A) Mice were treated with irradiation and lymphocyte infusion, and splenic dendritic cell (DC) (CD11c^+^MHCII^+^) percentages and CD86 expression were quantified by flow cytometry. B) Examination of inguinal lymph nodes (LN) for the percentages and relative ratios of skin-derived DCs (CD11c^+^MHCII^high^) and LN-resident DCs (CD11c^+^MHCII^intermediate^) was also evaluated by flow cytometry. Results shown from one of two experiments with similar results. C) Mice were treated with radiation and lymphocyte infusion, and were then immunized with hTRP2 DNA vaccine starting 1, 3, or 7 days afterwards. After 3 immunizations, splenocytes were restimulated with TRP2_181-188_ peptide or irradiated B16 cells, and IFNγ production from CD8^+^ T cells was quantified by flow cytometry. n=3/group, results shown from one of two experiments with similar results. D) Mice were treated as in A with varying days of initial hTRP2 DNA immunizations, and then were challenged intradermally with B16 melanoma. Mice were then monitored for development of palpable tumors. n=7-12/group, results shown from one of two experiments with similar results.

In addition to evaluating T cell changes in response to radiation, we also examined effects on dendritic cells (DCs), which are major mediators of T cell activation in response to tumor vaccination [20]. The first day after irradiation, we observed a series of dramatic changes in dendritic cell numbers and phenotype. The frequency of splenic DCs (CD11c^+^MHCII^+^) was increased (Figure 3A) and these DCs demonstrated higher expression of the costimulatory molecule CD86 (Figure 3A) as well as CCR7 and IL-15Rα (data not shown). We found similar results in inguinal lymph nodes (data not shown). In addition, in inguinal lymph nodes we observed an abrupt increase in the percentage of skin-derived DCs, which can be identified by a higher level of MHCII expression [21-24]. By 24 hours following irradiation, the percentage of skin-derived DCs increased greater than 10-fold. This was followed by a rapid decrease to a level that remained above normal for an additional 48 hours (Figure 3B). This influx of migratory DCs resulted in a higher ratio of MHCII^high^ to MHCII^intermediate^ DCs (Figure 3B). Migratory DCs have been previously shown to be superior in mediating T cell activation [21-24]. Together, these results suggest that radiation may mediate enhanced responses to tumor vaccination via improvements in antigen-presentation. However, by day 3, these changes had largely normalized and by day 5 following irradiation, DCs were nearly undetectable in peripheral lymphoid organs (Figure 3A).

Given the short-lived augmentation of dendritic cell numbers and activation phenotype, we evaluated whether the window of enhanced responsiveness to vaccination after irradiation is similarly short-lived. We treated mice with radiation followed by lymphocyte infusion, and initiated immunizations at varying time points thereafter. Mice vaccinated starting one day after treatment demonstrated enhanced T cell responses and tumor rejection, similar to what we had observed previously (Figures 3C and D). Interestingly, we found that delaying the start of vaccinations to either 3 days or 7 days after treatment led to a time-dependent loss of ability to respond to vaccination, with blunted T cell responses and impaired tumor rejection (Figures 3C and 3D). Together, these results suggest that radiation may function as an immune adjuvant via enhancement of antigen-presentation, but the effect is quite short-lived and the window of initiating effective immunization is less than 3 days in duration for this particular regimen.

Radiation followed by lymphocyte infusion improves the durability of T cell responses to tumor immunization and improves efficacy of tumor immunization as therapy for tumor-bearing mice and mice that develop spontaneous melanomas

**Figure 4:**
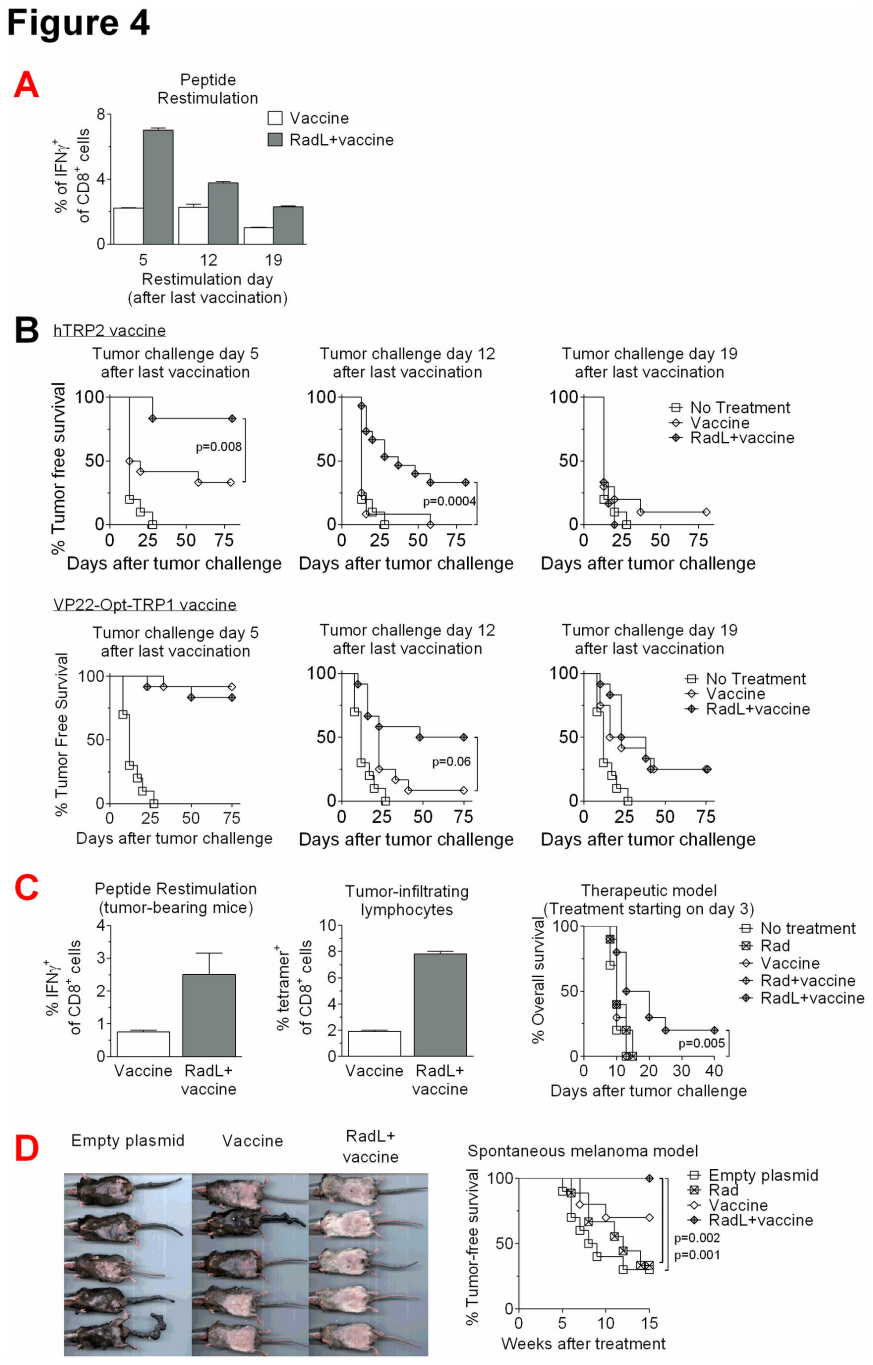
Radiation followed by lymphocyte infusion leads to improved persistence of responses to tumor vaccination and to cure of some mice with established tumors. A) Mice were treated with radiation and lymphocyte infusion, and were then immunized with VP22-Opt-TRP1 DNA vaccine starting 1 day afterwards for 3 immunizations. Either 5, 12, or 19 days after the last immunization, splenocytes were restimulated with TRP1_455-463_ peptide and IFNγ production from CD8^+^ T cells was quantified by flow cytometry. n=3/group, results shown from one of two experiments with similar results. B) Mice were treated as in A with hTRP2 or VP22-Opt-TRP1 DNA vaccine starting 1 day after radiation and lymphocyte infusion. Either 5, 12, or 19 days after the last immunization, mice were challenged intradermally with B16 melanoma. Mice were then monitored for development of palpable tumors. n=10-15/group, results shown from one of two experiments with similar results. C) Mice were inoculated intradermally with B16 melanoma. Three days later, some mice received radiation and lymphocyte infusion, followed 1 day later by immunizations with VP22-Opt-TRP1 DNA vaccine every 5 days. Mice were harvested on day 21 after irradiation and splenic and tumor-infiltrating lymphocytes were evaluated by flow cytometry. Additional mice were treated similarly for a total of 8 immunizations, n=10/group, and followed for overall survival, with results shown from one of three experiments with similar results. D) Tg(Grm1)EPv-transgenic mice began treatment at 8-12 weeks of age with 5 weekly vaccinations of VP22-Opt-TRP1 DNA or an empty control plasmid; some mice were pre-treated with irradiation and lymphocyte infusion one day prior. Mice were evaluated weekly for development of tail and ear melanomas. n=4-5/group, with combined results from 2 experiments.

It has been proposed that the degree of T cell memory development is correlated with the magnitude of initial immune responses [25,26]. We next examined the persistence of the augmented T cell responses following irradiation, infusion, and vaccination. We evaluated T cell responses of mice 5, 12, 19, and 35 days after completion of immunizations. We found that treating with radiation and lymphocyte infusion prior to vaccination led to increased T cell responses up to 19 days after the final immunization, though by day 35 these had waned to levels similar to unirradiated mice (Figure 4A). These results suggest that radiation and lymphocyte infusion not only increase peak T cell responses to immunization (day 5), but also led to a continued elevation in T cell responses, which lasted for several weeks. We hypothesized that this enhanced persistence of T cell responses would lead to improved rejection of tumor challenges administered at later time points after final immunization. We evaluated this with both hTRP2 and VP22-Opt-TRP1 and found that indeed, radiation and lymphocyte infusion improved tumor rejection at day 12 after final immunization (Figure 4B), though by day 19 this enhancement was no longer evident.

Given our overall findings demonstrating the potency of radiation and lymphocyte infusion as a tumor vaccine adjuvant, we evaluated the efficacy of this approach in tumor-bearing mice. We irradiated and vaccinated mice bearing 3-day-established B16 tumors, and one day later began immunizing with VP22-Opt-TRP1 plasmid DNA. We found that, similar to tumor-naïve mice, tumor-bearing mice develop improved responses to tumor vaccination following radiation and lymphocyte infusion, with enrichment of tumor-specific T cells in both the spleen and tumor (Figure 4C). Furthermore, when mice were followed for overall survival, we found that vaccination with VP22-Opt-TRP1 alone failed to cure mice bearing 3-day B16 tumors (Figure 4C), while the combination of vaccine, irradiation and lymphocyte infusion led to cures in 20% of mice (p=0.005, Figure 4C).

Finally, to better evaluate the potential of radiation as a cancer vaccine adjuvant in a clinically relevant model, we tested the efficacy of DNA vaccination in a spontaneous murine model of melanoma. Tg(Grm1)EPv-transgenic mice carry the metabotropic glutamate receptor 1 (Grm1) under the control of the melanocyte-specific dopachrome tautomerase (Dct, Trp2) promoter. These mice develop melanocytic hyperproliferation at hairless regions that progresses to distinct primary melanomas, with a latency of 4–6 months. We treated mice beginning at 8-12 weeks of age, and found that vaccination alone with VP22-Opt-TRP1 produced only partial protection in these mice, with 30% of mice succumbing to development of melanomas (Figure 4D). Radiation and lymphocyte infusion, however, improved the response to vaccination resulting in 100% tumor-free survival in this spontaneous melanoma model, which has a high penetrance without treatment. We also noted that mice vaccinated following radiation and lymphocyte infusion developed a higher degree of autoimmune-mediated vitiligo, compared to mice that were vaccinated without pretreatment (Figure 4D).

## Discussion

Prior studies have demonstrated that irradiation followed by lymphocyte infusion can enhance immune cell therapies against tumors [6-11]. In many of these studies, tumor antigens were either not well-defined or were genetically over-expressed foreign antigens. Also, tumor cell inoculation usually occurred prior to irradiation, leading to a potential contribution from immunogenic tumor cell death. In the current study, we sought to carefully define the mechanisms by which irradiation can act as a cancer vaccine adjuvant independent of immunogenic, irradiation-induced tumor cell death in a clinically relevant mouse model. We chose to immunize with DNA vaccines that elicit well-defined responses against tissue-restricted self-antigens, utilizing mice that had normal polyclonal T cell repertoires in tumor experiments, and evaluated responses against a pre-clinical mouse melanoma model.

We found that irradiation prior to vaccination resulted in enhanced responsiveness to plasmid DNA immunization against self-antigens, both in terms of quantified T cell responses and rejection of tumor cells. We demonstrated that several requirements are critical for the adjuvant effects of irradiation. First, a lymphocyte infusion following irradiation is necessary, because irradiation alone without lymphocyte infusion resulted in loss of responsiveness to DNA immunization. Our experiments utilizing congenic markers corroborated the importance of the lymphocyte infusion by demonstrating that T cells responding to vaccination were all derived from the infused lymphocytes and not from the irradiated mouse [27]. A recent clinical trial in patients with multiple myeloma also demonstrated the importance of lymphocyte infusion prior to immunization with a pneumococcal vaccine following myeloablative conditioning with melphalan [28].

Second, we found that the timing of immunizations was also crucial in determining whether irradiation could augment immune responses. Initiation of immunizations within 24 hours of irradiation led to augmented responses, but by 72 hours, the responsiveness of the immune system had waned to normal (unirradiated) levels. This was unlikely to be due to expansion of regulatory T cells or recovery from lymphopenia following irradiation, since our data demonstrate that depletion of all T cell subsets, including regulatory T cells, persists for up to 6 weeks following irradiation. Our results raise the intriguing possibility that enhancement of dendritic cell function following irradiation may in part account for the increased responsiveness of CD8+ T cells to vaccine. We observed a brief period of dendritic cell activation and recruitment following irradiation which rapidly returns to baseline by 72 hours after irradiation. If immunizations were initiated 24 hours after irradiation, then mice developed augmented acquisition of CD8+ T cell effector function, suggesting that priming by dendritic cells is enhanced in this time period. We previously demonstrated protein expression as early as 8 hours after DNA administration to the skin using the gene gun [29]. While the mechanism of increased DC migration to skin-draining lymph nodes after ionizing radiation remains unclear, acute radiation toxicity of the skin is associated with elevated levels of inflammatory cytokines and chemokines, including IL-1α, IL-1β, TNF-α, CCL4, CXCL10, and CCL2 [30-32]. Of the major groups of skin-derived DCs, both the epidermal LCs and the dermal DCs have been found to be depleted from the skin following local irradiation [33]. Our data identifying an increase in skin-derived DCs in the inguinal lymph nodes 24 hours following irradiation suggests that these skin DCs have indeed migrated to the draining lymph nodes, rather than dying off within the skin. Our data therefore suggest that early immunization allows for antigen uptake by enriched and activated DCs that can more effectively present to the immune system. Interestingly, our data also show that the immune advantages conferred by irradiation do not result from an increase in higher affinity T cells but rather an increase in either recruitment or proliferation of low-affinity T cells. Thus enhanced antigen-presentation may indeed be playing a dominant role in augmenting immune responses by improving activation of low-affinity T cells.

In contrast to DCs, T cell populations were initially depleted after irradiation and then gradually recovered over 6 weeks. It has been suggested that CD8+ T cells may expand more rapidly than regulatory T cells in the lymphopenic host and that this may result in enhanced effector function [12,34]. In our model, we found that numbers of CD4+Foxp3+ T cells and CD8+ T cells are both significantly depleted after irradiation. In the first few days after irradiation, however, CD8+ T cells recovered more robustly, leading to a dramatic increase in the ratio of donor-derived CD8+ T cells to CD4+Foxp3+ T cells. This may be due to an augmentation in CD8+ T cells responsiveness to IL-15 following irradiation; indeed, we observed an upregulation of the components of the IL-15 receptor including IL-15Rα and CD122. One potential mechanism for increased expression of IL-15 receptor components in lymphopenia may be through reduced TGFβ signaling, since TGFβ has been known to modulate expression of CD122 [35]. IL-15 signaling has been shown to promote CD8+ T cell survival in the lymphopenic environment [10]. Our results corroborate studies in both mouse models [36] and clinical studies [37], which indicate that the effector to regulatory T cell ratio can be a useful predictor for development of effective anti-tumor immune responses. 

Our data suggest that irradiation can augment immune responses to cancer vaccines that already contain additional immune adjuvants. In our experiments we utilized DNA vaccines, which are bacterial plasmids and thus include CpG motifs that signal through TLR9 [38]. In our tumor models, however DNA vaccination alone provided only partial protection which could be enhanced be pre-treating mice with radiation and lymphocyte infusion prior to vaccination. Our data thus indicate that irradiation can act as an immune adjuvant even in the presence of another adjuvant, such as CpG.

While our study addressed tumor-independent immune effects of irradiation, tumor-specific immune effects of irradiation have been demonstrated by others. These include antigen release and cytokine production, which results in increases in tumor infiltration by lymphocytes and enhanced anti-tumor immune responses [39,40]. External beam radiation applied to tumors can augment effects of vaccines [41], as well as other immunomodulating strategies, including CTLA-4 and PD-1 blockade [42,43]. One study demonstrated the generation of tumor-specific T cell immune responses after irradiation [44], and showed that these cells affect not only the locally irradiated area, but also distant metastatic sites. Recent clinical data suggests that this can occur in humans as well [45]. The response seems to depend on the dose and frequency of radiation (single dose versus fractionated) [43,44]. Thus, more than a single mechanism likely accounts for the immunostimulatory effect of irradiation.

We sought to evaluate the clinical potential of utilizing irradiation as an immune adjuvant. We found that irradiation and lymphocyte infusion led to not only higher peak responses, but also improved persistence of immune responses, measured by both T cell stimulation assays and tumor challenge experiments. Importantly, we found that radiation and lymphocyte infusion acts as a tumor vaccine adjuvant in both the absence and presence of tumor. Interestingly, our data suggest that the presence of aberrant melanocyte proliferation may be important in generating lasting anti-tumor immunity. While wild-type mice generate robust T cell responses to DNA vaccination that are further enhanced when mice are pre-treated with radiation and lymphocyte infusion, immune responses fade over time and mice become susceptible to tumor challenges at later time points. In a spontaneous model of murine melanoma, however, radiation followed by lymphocyte infusion and vaccination produces lasting protection against spontaneous development of melanomas. It is possible that melanocyte hyperproliferation generates a broad immune response against a variety of antigens, and that in this setting generating an immune response against single self-antigen can lead to lasting protection.

In summary, irradiation in an appropriate time frame can act as a potent immune adjuvant for DNA immunization against self-derived differentiation antigens, leading to augmented anti-tumor immunity. Essential requirements that we have identified include lymphocyte infusion to rescue immune responsiveness and early initiation of vaccination. Our data raise the hypothesis that transient enhancement of dendritic cell function may be responsible for the augmented CD8+ T cell response to vaccination. These results have important implications for the design of clinical trials utilizing combination strategies of radiation therapy and immunotherapy in patients with cancer.

## Materials and Methods

### Mice and adoptive transfer

C57BL/6J (B6, H-2b) and congenic B6 Thy1.1 mice (6-8 week-old females) were obtained from the Jackson Laboratory (Bar Harbor, ME). Thy1.1+ Pmel T cell receptor transgenic (Tg) mice have been reported and were kindly provided by Nicholas Restifo, National Cancer Institute, Bethesda, MD [18]. For adoptive transfer experiments, splenocytes from naïve B6 Thy1.1 mice were injected by tail vein into B6 Thy1.2 recipients 1-2 hours after 600 cGy total body irradiation from a ^137^Cs source. Mouse studies were approved by the Memorial Sloan-Kettering Cancer Center Institutional Animal Care and Use Committee. 

### Plasmid DNA constructs

Human TRP2 (hTRP2) was cloned into the pCR3 vector, with a CMV promoter and ampicillin-resistance gene, as previously described [16]. The human gp100 (hgp100) expression vector contains full length hgp100 cDNA cloned into the WRG/BEN vector, with a CMV promoter and kanamycin-resistance gene [46,47]. VP22-Opt-TYRP1 DNA was constructed by optimizing the coding sequence of mouse TYRP1 for MHC class I binding to both Kb and Db and fusing it to VP22, an HSV-1 protein that has been shown to enhance vaccine potency [19,27,48]. We have previously shown that immunization with the plasmid vector alone did not induce tumor rejection or antigen-specific responses [16,46,49]. 

### Plasmid DNA vaccine administration

Mice were immunized by helium-driven particle bombardment, as previously reported [50]. Briefly, plasmid DNA was purified and coated onto 1.0 µm-diameter gold particles (Alfa Aesar, Ward Hill, MA) and precipitated on bullets of Teflon tubing. Gold particles containing 1 µg of DNA were delivered to each abdominal quadrant using a helium-driven gene gun (Accell; PowderMed, Oxford, United Kingdom), for a total of 4 µg of DNA per mouse. 

### Mouse tumor studies

Tumor challenge experiments were carried out with melanoma B16 cells, as described previously [51]. Briefly, 5×10^4^ B16F10 (B16) melanoma cells (gift of Isaiah Fidler, MD Anderson Cancer Center, Houston, TX) were injected into the shaved right flank of the mice. Tumor diameters were measured by calipers every 2-3 days, and mice were sacrificed when diameter exceeded 1 cm, tumors became ulcerated, or mice showed discomfort. Tumor-free survival was assessed from the day of tumor challenge. Kaplan-Meier survival curves were generated and compared using the log-rank test. For tumor harvest experiments, mice were challenged with 25×10^4^ B16 melanoma cells in Matrigel injected subcutaneously 3 days prior to irradiation and adoptive transfer.

### Dendritic cell preparation

Cells from draining lymph nodes or spleens were digested by treatment with collagenase D (1 mg/mL; Roche, Indianapolis, IN) and DNase I (2 mg/mL; Sigma, St Louis, MO) for 60 minutes at 37°C. After washing, the cell suspension was prepared.

### Antibodies and flow cytometry

Anti–murine CD16/CD32 FcR block (2.4G2) and all of the following fluorochrome-labeled antibodies against murine antigens were obtained from BD PharMingen (San Diego, CA): CD3 (145-2C11), CD4 (RM4-5), CD8 (53-6.7), CD62L (MEL-14), CD122 (TM-B1), CD44 (IM7), CD107a (1D4B), Thy1.1 (OX-7), CD45R/B220 (RA3-6B2), NK1.1 (PK136), CD11b (M1/70), CD25 (PC61), CD69 (H1.2F3), IFNγ (clone XMG1.2); isotype controls: rat IgG2a-κ (R35-95), rat IgG2a-λ (B39-4), rat IgG2b-κ (A95-1), rat IgG1-κ (R3-34), hamster IgG–group1-κ (A19-3), hamster IgG–group 1-κ (Ha4/8), and streptavidin-FITC, -PE, and -PerCP. Additional anti-mouse antibodies against IL-15Rα (R&D Systems, Minneapolis, MN), CD62L (Invitrogen, Carlsbad, CA), Foxp3 (eBioscience, San Diego, CA) human Fcγ-specific (Jackson ImmunoResearch, West Grove, PA) antibodies. FACS staining was performed as previously described [52]. Cells were acquired on a FACSCalibur or LSR II cytometer (Becton Dickinson, San Jose, CA) with CellQuest software. Data were analyzed with FlowJo software (Treestar, San Carlos, CA). 

### T cell assays

For all assays, spleens and draining lymph nodes were harvested from mice (3-5/group), pooled within groups, crushed and filtered through 0.22 μm cell strainers. Red blood cells were lysed using an ammonium chloride lysis buffer. Cells were washed twice in RPMI + 7.5% fetal calf serum prior to assays. Samples were run in singlet, duplicate, or triplicate depending on number of cells available, and results were averaged, with error bars indicating standard errors.

Tetramer assay: PE-conjugated TRP1_455_-Db-tetramer, containing the Db epitope (TAPDNLGYM) [19] and PE-conjugated hgp100_25-33_-Db-tetramer, containing the Db epitope KVPRNQDWL [46,47] were from Beckman Coulter. For tetramer production, the optimized (TRP1_455_) or human (gp100_25_) sequences were used rather than the native mouse sequences since only the higher affinity peptides were able to form tetramers. 

Intracellular cytokine assay: The procedure was performed as previously described [27]. Unselected cells (5×10^6^/well) were stimulated for 16 hours with irradiated B16 or with 1 μg/mL peptide in the presence of irradiated EL4 cells, at a ratio of 5:1. Brefeldin A (10 μg/mL, Sigma, St. Louis, MO) was added one hour after the peptide. Following stimulation, cells were stained for surface markers and intracellular IFNγ using the Cytofix/Cytoperm Kit (BD PharMingen) according to the manufacturer's instructions and analyzed on a flow cytometer. 

### Spontaneous melanoma model

Tg(Grm1)EPv-transgenic mice carry the metabotropic glutamate receptor 1 (Grm1) under the control of the melanocyte-specific dopachrome tautomerase promoter[53]. Mice develop melanocytic hyperproliferation at hairless regions that progress to distinct primary melanomas, with a latency of 4–6 months and nearly 100% penetrance. Mice began treatment at 8-12 weeks of age and were treated with 5 weekly vaccinations.
